# The Relationship Between Critical Thinking Skills and Learning Styles and Academic Achievement of Nursing Students

**DOI:** 10.1097/jnr.0000000000000307

**Published:** 2019-07-16

**Authors:** Fatemeh Shirazi, Shiva Heidari

**Affiliations:** 1 PhD, Assistant Professor, Department of Nursing, School of Nursing and Midwifery, Shiraz University of Medical Sciences, Shiraz, Iran; 2 MSN, Instructor, Department of Nursing, Urmia Branch, Islamic Azad University, Urmia, Iran.

**Keywords:** academic achievement, critical thinking skills, learning styles, nursing student

## Abstract

**Background:**

Academic achievement is one of the most important indicators in evaluating education. Various factors are known to affect the academic achievement of students.

**Purpose:**

This study was performed to assess the relationship between critical thinking skills and learning styles and the academic achievement of nursing students.

**Methods:**

In this cross-sectional study, 139 sophomores to senior-year nursing students were selected using a simple random sampling method. The data were gathered using a three-part questionnaire that included a demographic questionnaire, the Kolb's Learning Style Standard Questionnaire, and the California Critical Thinking Skills Questionnaire. The previous semester's grade point average of the students was considered as a measure of academic achievement. The data were analyzed using descriptive and analytical statistics in SPSS 20.

**Results:**

The mean score for critical thinking skills was 6.75 ± 2.16, and the highest and lowest scores among the critical thinking subscales related to the evaluation and analysis subscales, respectively. No relationship between critical thinking and academic achievement was identified. “Diverging” was the most common learning style. The highest mean level of academic achievement was earned by those students who adopted the “accommodating” style of learning. A significant relationship was found between learning style and academic achievement (*p* < .001).

**Conclusions:**

According to the findings, the critical thinking skills score of students was unacceptably low. Therefore, it is essential to pay more attention to improving critical thinking in academic lesson planning. As a significant relationship was found between learning style and academic achievement, it is suggested that instructors consider the dominant style of each class in lesson planning and use proper teaching methods that take into consideration the dominant style.

## Introduction

Academic achievement is crucial to the future success of students, and lack of attention to this basic issue and subsequent academic failure may cause a decrease in academic accomplishment and an increase in the costs of education (Jayanthi, Balakrishnan, Ching, Latiff, & Nasirudeen, 2014). Academic achievement, the level to which students attain predetermined educational goals, depends on family and individual, socioeconomic, education, training, and psychological factors (Farooq, Chaudhry, Shafiq, & Berhanu, 2011). Assessing these factors and determining the contribution of each to academic achievement are critical to developing strategies for identifying the factors that contribute to academic success and failure and help educational planners focus on promoting the positive factors and reducing the impact of negative factors (Gordon, Williams, Hudson, & Stewart, 2010). Critical thinking is one of the contributing factors in academic achievement as well as an essential component in clinical decision making, nursing practice, and education (Fero et al., 2010).

There are many reasons for nurses to learn critical thinking skills. The first reason is that thinking is the key component in problem solving, and nurses without these proficiencies become part of the problem. In addition, nurses should be capable of making major decisions independently and quickly in critical situations. Critical thinking skills enable them to identify essential data and distinguish between problems that require urgent intervention and those that are not life-threatening. Thus, nurses should be able to reflect on their actions and consider the possible consequences of each action to make precise and proper decisions (Eslami & Maarefi, 2010).

Various investigations have suggested that it is necessary to design educational strategies that are based on student learning style to improve students' critical thinking. In addition to critical thinking, the learning styles of students are an important factor that plays a fundamental role in the process of problem solving and learning. Learning style describes the method used to process information, which differs from person to person. Identifying the methods that students use to process information and their learning styles allows educators to assist them to advance toward the higher goals of training and achieve broader critical thinking and problem-solving skills (Lau & Yuen, 2010). Perhaps, the best definition of learning styles was provided by Kolb, who defined learning styles as an individual's method of emphasizing certain learning abilities over other abilities. Kolb's experiential learning theory is the result of the combination of three templates from the experiential learning process, including Lewin's practical and laboratory model, Dewey's learning model, and Piaget's pattern of learning and cognitive development. Kolb believed learning to be the result of resolving the conflicts among these three models (Kolb & Kolb, 2005). Many studies have investigated the relationships between learning styles and other variables. The academic achievement of learners is one of the key variables to be studied with regard to its relationship with learning style (Zainol Abidin, Rezaee, Abdullah, & Singh, 2011). Most of these studies have shown thinking to be the combination of knowledge, skills, and attitudes. This combination empowers thoughtful persons to become wiser and more competent in different sciences and technologies and, consequently, gives them momentum along the path to success (Can, 2009). The study of Aripin, Mahmood, Rohaizad, Yeop, and Anuar (2008) showed that paying attention to students' learning styles and matching these with a learning framework significantly improved students' academic performance, whereas a mismatch between learning styles and curriculum reduced performance levels. Other studies that surveyed the relationship between critical thinking and its subdomains and different learning styles obtained different results (Ghazivakili et al., 2014; Noohi, Salahi, & Sabzevari, 2014).

The review of studies highlighted conflicting results in the relationship between critical thinking, learning styles, and academic achievement. Some studies have emphasized a positive relationship (Ashoori, 2014; Ghazivakili et al., 2014), whereas others have found a negative relationship (Aghaei, Souri, & Ghanbari, 2012) or an absence of a significant relationship. These conflicting results may be caused by differences among individual student characteristics and their educational culture (Abdollahi Adli Ansar, FathiAzar, & Abdollahi, 2015). On the basis of these differences in results and the diversity of students and educational systems in different academic contexts, this study was designed to determine the relationship between critical thinking skills and learning styles and the academic achievement of nursing students studying at Urmia Islamic Azad University.

## Methods

In this cross-sectional study, 139 nursing students between their sophomore and senior years were selected randomly out of 360 nursing students studying at Islamic Azad University in Urmia, Iran. The students were divided into three groups according to their years of education, and each group was random sampled using a table of random numbers. The researcher delivered the questionnaires and consent forms to the selected students. After explaining how to answer the questions, the completed questionnaires were collected 2 days later. Data collection lasted from October to December 2015.

This study was approved by the research council and the ethics committee of the Urmia branch of Islamic Azad University, Urmia, Iran (Code: 27827). A three-part questionnaire was used for data collection. The first part of the questionnaire assessed demographic information such as age, marital status, and educational level. Besides that, the grade point average (GPA) of each student for the previous semester was recorded as a measure of academic achievement. The second part of the questionnaire was California Standardized Critical Thinking Skills Test, Form B, published by Facione and Facione in 1994. This test contains 34 multiple (4–5)-choice questions with one correct answer each. These questions address the five domains of the cognitive skills of critical thinking (deductive, inductive, assessment, analysis, and inference). One score is assigned for each correct answer, and the total test score is obtained by summing the number of correct answers. The minimum and maximum possible scores are 0 and 34, respectively. The midpoint score of the scale is 15.98, indicating that lower scores represent relatively weak critical thinking and higher scores represent relatively strong critical thinking. The reliability of this questionnaire was reported as .86 by Hariri (Hariri & Bagherinejad, 2012). In this study, the reliability of the test was checked using test–retest, with an earned score of .79.

The third part of the questionnaire was Kolb's Learning Styles Inventory, which includes 12 sentences. Each sentence includes four parts that respectively measure reflective observation, concrete experience, active experimentation, and abstract conceptualization. The four scores obtained from the sum of these four parts in the 12 questions of the questionnaire indicate the four styles of learning. Two scores are obtained from two-by-two subtraction of these styles, that is, the subtraction of abstract conceptualization from concrete experience and active experimentation from reflective observation. These two scores are placed on the axis, which constitutes the four quarters of a square, identified by the four learning styles as diverging, converging, assimilating, and accommodating (Kolb & Kolb, 2005). Emamipour reported the alpha coefficients of abstract conceptualization, concrete experience, active experimentation, and reflective observation as .49, .51, .47, and .53, respectively (Emamipour & Shams Esfandabad, 2007).

After collecting the completed questionnaires, the data were analyzed using SPSS software Version 20 with descriptive and analytical statistical tests such as Student *t* test, one-way analysis of variance, chi-square, and correlation test.

## Results

The data analysis showed that all of the participants were female, with a mean age of 21.88 ± 2.09 years and an age range of 19–29 years. Most (85.6%) were single (Table 1). The mean GPA of the students was 15.78 ± 1.35, ranging from 12 to 18.79.

**TABLE 1.**
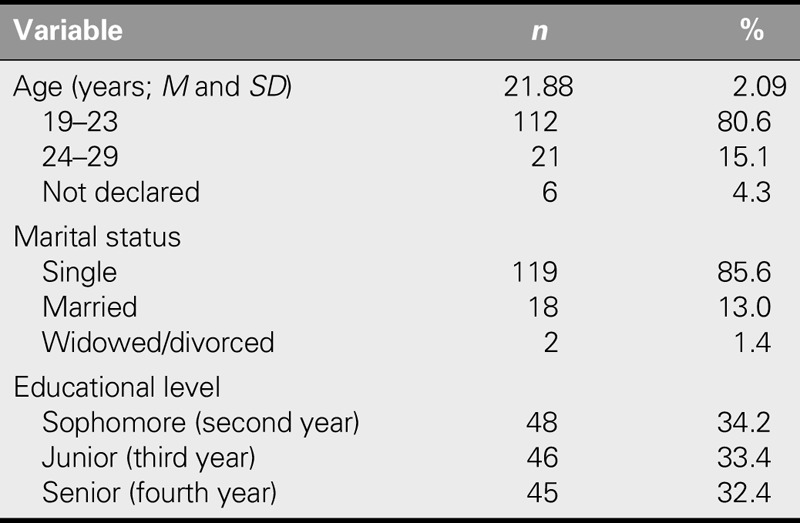
Demographic Characteristics of Participants (*N* = 139)

The mean score of critical thinking was 6.75 ± 2.16, and the highest and lowest mean critical thinking skill subdomain scores were for evaluation skill (6.75 ± 2.16) and analysis skill (1.58 ± 1.85), respectively. No significant relationship between critical thinking and academic achievement was found. Moreover, the critical thinking subdomains were not significantly related to academic achievement. In addition, no significant relationship was found between the total score and the subscales of critical thinking and marital status, age, or educational level. However, a significant relationship was found between the total score of critical thinking and educational level. Therefore, the senior students in this study earned a higher mean score for critical thinking than their lower-grade peers (*p* = .04; Table 2).

**TABLE 2.**
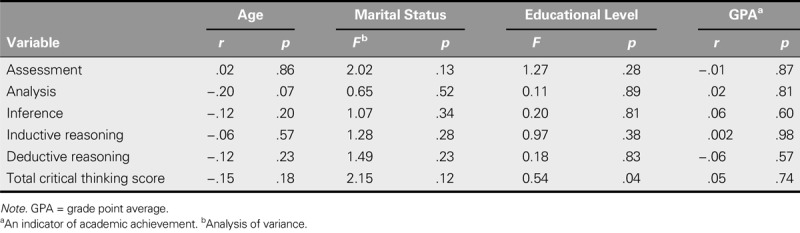
The Relationship Between Critical Thinking Styles and Demographic Variables and Academic Achievement

Most participants (55.4%) used a “diverging” learning style, whereas 0.7% used a “converging” style. There was a significant relationship between learning styles and academic achievement, with academic achievement (represented by GPA) highest in the accommodating learning style subgroup followed by the diverging, converging, and assimilating learning-style subgroups.

Whereas no significant relationship was found between learning style and either age or educational level, a significant relationship was found between learning style and marital status (Table 3).

**TABLE 3.**
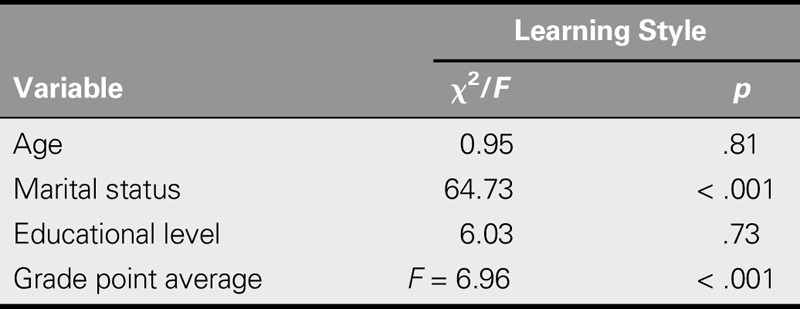
Relationship Between Learning Style and Demographic Variables and Academic Achievement

Using one-way analysis of variance, the relationship between learning style and critical thinking skills and also the comparison of the mean score for each skill in four styles are reported in Table 4. The findings showed no statistically significant relationship between learning style and critical thinking or its subscales (Table 4).

**TABLE 4.**
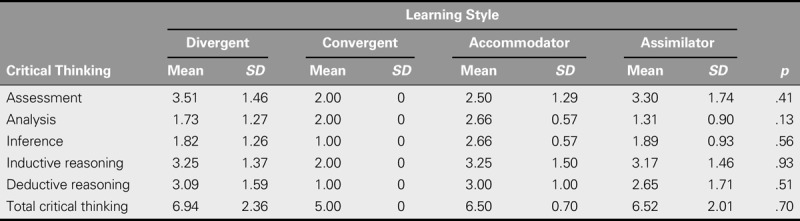
Relationship Between Critical Thinking Skills and Learning Style

Finally, a significant relationship was found between academic achievement and educational level, which meant that senior students had the highest level of academic achievement (*p* = .01). However, academic achievement had no significant relationship with other variables such as age and marital status (Table 5).

**TABLE 5.**
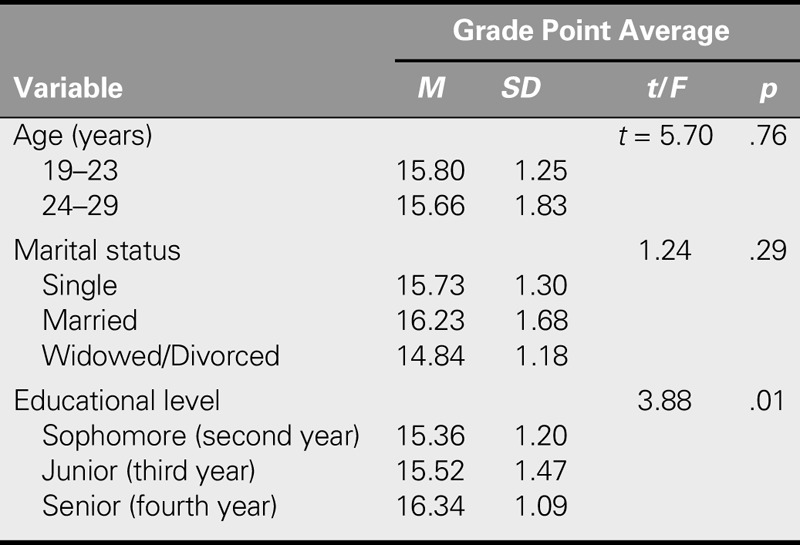
Relationship Between Demographic Variables and Academic Achievement

## Discussion

This study found no significant relationship between any of the demographic variables such as age and marital status and academic achievement. However, years of education were associated positively with academic achievement. This finding is consistent with that of Edraki, Rambod, and Abdoli (2011). Fewer courses in higher academic grades, familiarization with the university atmosphere, and the stronger emphasis on clinical courses during the years of education may help students to effectively increase their GPA and improve their academic achievements.

The mean score for critical thinking in this study was 6.75 ± 2.16. Similar to the results of this study, Taghavi Larijani, Mardani Hmouleh, Rezaei, Ghadiriyan, and Rashidi (2014) reported a mean score for critical thinking of 9.33 ± 3.33. In addition, a study conducted in the United States found that most students earned relatively low scores for critical thinking (Shinnick & Woo, 2013). On the contrary, a 2010 study in Norway found that participants earned good scores for critical thinking (Wangensteen, Johansson, Björkström, & Nordström, 2010). Researchers believe that the multiple, intertwining factors involved in decreasing critical thinking scores include educational failure, focusing on rote memorization, presenting concepts in manners that do not require deep questioning/consideration, emphasis on multiple-choice examinations, lack of appropriate mental or psychological security for questioning and answering between the students and instructors, and poor development of critical thinking abilities (Hosseini, 2009). The low critical thinking score of the students in this research as well as in other studies conducted in Iran compared with the scores of students in other countries suggests that current education methods in Iran do not effectively strengthen the critical thinking of students and thus should be revised (Azodi, Jahanpoor, & Sharif, 2010).

In addition, in this study, the maximum and minimum subdomain scores for critical thinking were for assessment and analysis, respectively. Similarly, Ghazivakili et al. found that the minimum score for critical thinking was in the dimension of analysis (Ghazivakili et al., 2014). On the basis of the findings of this study, no significant relationship was observed between the critical thinking and the academic achievement of the students, which is consistent with the results of Azodi et al. (2010). Furthermore, the findings of Ghazivakili et al. suggested a relationship between critical thinking skills and the previous semester's GPA as a criterion for determining academic achievement. In the study of Ghazivakili et al., the mean GPA score of the students was increased by increasing the understanding skill of critical thinking (Ghazivakili et al., 2014).

This study did not show any relationship between critical thinking and either age or marital status. However, Azodi et al.'s study showed a positive relationship between age and critical thinking (Azodi et al., 2010). Age is an important demographic variable that is often correlated with critical thinking. This relationship is based on the assumption that critical thinking improves with age (Babamohammadi, Esmaeilpour, Negarande, & Dehghan Nayeri, 2011).

The relationship between the total score for critical thinking and educational level was significant. Thus, the total score for critical thinking increased with the number of years of enrollment. However, no relationship was observed between the subdomains of critical thinking and educational level, which is consistent with the findings of Noohi et al. (2014).

The results showed that diverging, assimilating, accommodating, and converging were, respectively, the most-to-least used learning styles of the participants in this study. This ranking of students' learning styles differs from those of other studies that were conducted domestically and outside Iran. Most participants adopted the assimilating learning style in the research of Tulbure (2012), whereas Orhun (2012) found that most participants preferred the converging learning style. This variation may reflect differences in educational settings and/or educational methods.

It seems that the diverging learning style is more appropriate for the field of nursing due to the nature of the field and the career prospects of nursing and midwifery students (Ahanchian, Mohamadzadeghasr, Garavand, & Hosseini, 2012). This learning style encourages students to be holistic and sociable; to use their ingenuity and thoughts in social situations and communication, especially with patients; and to be creative learners. These students develop and implement creative, workable, and effective solutions when dealing with complex patient issues and instill strong problem-solving capabilities. Thus, it is better to select those students who have diverging and accommodating learning styles for the field of nursing (Mohammadi, Sayehmiri, Tavan, & Mohammadi, 2013).

In determining the relationship between learning style and academic achievement, the results showed a significant relationship between these two variables. Thus, the highest average of academic achievement was earned, in rank order, by students who used accommodating, diverging, converging, and assimilating learning styles. A relationship between learning styles and academic achievement has also been suggested by Ahadi, Abedsaidi, Arshadi, and Ghorbani (2010) and Ghazivakili et al. (2014), but not by Farmanbar, Hosseinzadeh, Asadpoor, and Yeganeh (2013). The accommodating learning style is created from the combination of active experimentation and concrete experience. Users of this style learn and enjoy through practical work, work on projects, and engage in new tasks and controversial experiences. Preferred methods for accommodators include role playing and computer simulations. Accommodators have a tendency to engage in experimental work and to use various methods to achieve a goal (Pazargadi &Tahmasebi, 2010).

From the perspective of Kolb, learning style is a combination of cognitive, affective, and psychological properties. People advance their knowledge based on their learning style that has a significant role in their academic achievement. People have their own style of learning. Therefore, if the learning strategies of an individual match his or her learning style, performance is expected to improve (Panahi, Kazemi, & Rezaie, 2012).

Comprehending the learning styles of students is crucial for teachers, because each learning style requires the provision of appropriate educational materials (Gurpinar, Alimoglu, Mamakli, & Aktekin, 2010). The alignment of instructors' teaching styles to students' learning styles results in improved student understanding (Mlambo, 2011).

In surveying the relationship between critical thinking and learning styles, the critical thinking score was not statistically different among the four learning-style groups. Nevertheless, the results showed that the mean scores for critical thinking skill were found, from highest to lowest, in the diverging, assimilating, accommodating, and converging learning-style groups. In terms of the subscales, the highest average score was “assessment” in the diverging style group. The results of Noohi et al. also showed a higher score for critical thinking among converging people than among assimilating, accommodating, and diverging people (Noohi et al., 2014). Unlike the finding of this study, Ghazivakili et al. found that the total score of critical thinking differed among the four learning-style groups and that two of the subscales of critical thinking (evaluation and inductive reasoning) were positively related to learning styles (Ghazivakili et al., 2014).

Whereas no significant relationship was observed between learning style and either age or educational level in this study, significant relationships were found in the study of Ghazivakili et al. (2014). Furthermore, whereas both this study and Ahadi et al.'s (2010) study found a positive relationship between marital status and learning styles, Ghazivakili et al. reported no relationship with these two variables.

### Conclusions

The findings show that the mean scores of critical thinking skills and its subdomains were low among the nursing students who were surveyed for this study. Some strategies that may be used to improve critical thinking in this population include frequent use of individual and group active learning strategies, empowering instructors to prepare tests that target high levels of cognitive domain and present probing questions, encouraging students and instructors to participate in problem analysis and discussions, providing different ideas and opinions, and promoting self-directed learning (Shirazi, Sharif, Molazem, & Alborzi, 2016). It is hoped that the findings of this research attract the attention of instructors and managers regarding the importance of critical thinking evaluation in students. In addition, obtaining information about the dominant learning styles of students may encourage and enable nursing instructors to create appropriate learning environments and prepare the areas for academic achievement of the students. Learning outcomes improve when training matches the learning styles of the students.

## References

[bib1] Abdollahi Adli Ansar V. FathiAzar A. & & Abdollahi N. (2015). The relationship of critical thinking with creativity, self-efficacy beliefs and academic performance of teacher–students. *Journal of Research in School and Virtual Learning*, 2(7), 41–52. (Original work published in Persian)

[bib2] Aghaei N. Souri R. & & Ghanbari S. (2012). Comparison of the relationship between critical thinking and academic achievement among physical education students and students in other fields of study in Bu Ali Sina University, Hamedan. *Management of Sport and Movement Sciences*, 2(4), 35–45. (Original work published in Persian)

[bib3] Ahadi F. Abedsaidi J. Arshadi F. & & Ghorbani R. (2010). Learning styles of nursing and allied health students in Semnan University of Medical Sciences. *Koomesh*, 11(2), 141–146. (Original work published in Persian)

[bib4] Ahanchian M. Mohamadzadeghasr A. Garavand H. & & Hosseini A. (2012). Prevalent learning styles among nursing and midwifery students and its association with functionality of thinking styles and academic achievement a study in Mashhad School of Nursing and Midwifery. *Iranian Journal of Medical Education*, 12(8), 577–588. (Original work published in Persian)

[bib5] Aripin R. Mahmood Z. Rohaizad R. Yeop U. & & Anuar M. (2008). Student learning styles and academic performance. Paper presented at the Proceedings of the 22nd Annual SAS Malaysia Forum, Malaysia.

[bib6] Ashoori J. (2014). Relationship between self-efficacy, critical thinking, thinking styles and emotional intelligence with academic achievement in nursing students. *Scientific Journal of Hamadan Nursing & Midwifery Faculty*, 22(3), 15–23. (Original work published in Persian)

[bib7] Azodi P. Jahanpoor F. & & Sharif F. (2010). Critical thinking skills of students in Bushehr University of Medical Sciences. *Interdisciplinary Journal of Virtual Learning in Medical Sciences*, 1(2), 10–16. (Original work published in Persian)

[bib8] Babamohammadi H. Esmaeilpour M. Negarande R. & & Dehghan Nayeri N. (2011). Comparison of critical thinking skills in nursing students of Semnan and Tehran Universities of Medical Sciences. *Journal of Rafsanjan University of Medical Sciences*, 10(1, Suppl.), 67–78. (Original work published in Persian)

[bib9] Can Ş. (2009). The effects of science student teachers' academic achievements, their grade levels, gender and type of education they are exposed to on their 4mat learning styles (Case of Muğla University, Turkey). *Procedia-Social and Behavioral Sciences*, 1(1), 1853–1857. 10.1016/j.sbspro.2009.01.327

[bib10] Edraki M. Rambod M. & & Abdoli R. (2011). The relationship between nursing students' educational satisfaction and their academic success. *Iranian Journal of Medical Education*, 11(1), 32–39. (Original work published in Persian)

[bib11] Emamipour S. & & Shams Esfandabad H. (2007). Learning and cognitive styles: Theories and tests (pp. 453–472). Tehran, Iran: Samt Publisher (Original work published in Persian)

[bib12] Eslami A. R. & & Maarefi F. (2010). A comparison of the critical thinking ability in the first and last term baccalaureate students of nursing and clinical nurses of Jahrom University of Medical Sciences in 2007. *Journal of Jahrom University of Medical Sciences*, 8(1), 37–45. (Original work published in Persian)

[bib13] Facione P. A. & & Facione N. (1994). *The California Critical Thinking Skills Test: Test manual*. Millbrae, CA: California Academic Press.

[bib14] Farmanbar R. Hosseinzadeh T. Asadpoor M. & & Yeganeh M. (2013). Association between nursing and midwifery students' learning styles and their academic achievements, based on Kolb's model. *Journal of Guilan University of Medical Sciences*, 22(86), 60–68. (Original work published in Persian)

[bib15] Farooq M. S. Chaudhry A. H. Shafiq M. & & Berhanu G. (2011). Factors affecting students' quality of academic performance: A case of secondary school level. *Journal of Quality and Technology Management*, 7(2), 1–14.

[bib16] Fero L. J. O'Donnell J. M. Zullo T. G. Dabbs A. D. Kitutu J. Samosky J. T. & & Hoffman L. A. (2010). Critical thinking skills in nursing students: Comparison of simulation-based performance with metrics. *Journal of Advanced Nursing*, 66(10), 2182–2193. 10.1111/j.1365-2648.2010.05385.x 20636471 PMC5939573

[bib17] Ghazivakili Z. Norouzi Nia R. Panahi F. Karimi M. Gholsorkh H. & & Ahmadi Z. (2014). The role of critical thinking skills and learning styles of university students in their academic performance. *Journal of Advances in Medical Education & Professionalism*, 2(3), 95–102. 25512928 PMC4235550

[bib18] Gordon C. D. Williams S. K. P. Hudson G. A. & & Stewart J. (2010). Factors associated with academic performance of physical therapy students. *West Indian Medical Journal*, 59(2), 203–208. 21275127

[bib19] Gurpinar E. Alimoglu M. K. Mamakli S. & & Aktekin M. (2010). Can learning style predict student satisfaction with different instruction methods and academic achievement in medical education? *Advances in Physiology Education*, 34(4), 192–196. 10.1152/advan.00075.2010 21098386

[bib20] Hariri N. & & Bagherinejad Z. (2012). Evaluation of critical thinking skills in students of health faculty, Mazandaran University of Medical Sciences. *Journal of Mazandaran University of Medical Sciences*, 21(1), 166–173. (Original work published in Persian)

[bib21] Hosseini Z. (2009). Cooperative learning and critical thinking. *Developmental Psychology (Journal of Iranian Psychologists)*, 5(19), 199–208. (Original work published in Persian)

[bib22] Jayanthi S. V. Balakrishnan S. Ching A. L. S. Latiff N. A. A. & & Nasirudeen A. M. A. (2014). Factors contributing to academic performance of students in a tertiary institution in Singapore. *American Journal of Educational Research*, 2(9), 752–758. 10.12691/education-2-9-8

[bib23] Kolb A. Y. & & Kolb D. A. (2005). *The Kolb learning style inventory—Version 3.1 2005 technical specifications*. Boston, MA: Hay Resources Direct.

[bib24] Lau W. W. F. & & Yuen A. H. K. (2010). Gender differences in learning styles: Nurturing a gender and style sensitive computer science classroom. *Australasian Journal of Educational Technology*, 26(7), 1090–1103. 10.14742/ajet.1036

[bib25] Mlambo V. (2011). An analysis of some factors affecting student academic performance in an introductory biochemistry course at the University of the West Indies. *Caribbean Teaching Scholar*, 1(2), 79–92.

[bib26] Mohammadi I. Sayehmiri C. Tavan H. & & Mohammadi E. (2013). Learning styles of Iranian nursing students based on Kolb's theory: A systematic review and meta-analysis study. *Iranian Journal of Medical Education*, 13(9), 741–752. (Original work published in Persian)

[bib27] Noohi E. Salahi S. & & Sabzevari S. (2014). Association of critical thinking with learning styles in nursing students of school of nursing and midwifery, Iran. *Journal of Strides in Development of Medical Education*, 11(2), 179–186. (Original work published in Persian)

[bib28] Orhun N. (2012). The relationship between learning styles and achievement in calculus course for engineering students. *Procedia-Social and Behavioral Sciences*, 47, 638–642. 10.1016/j.sbspro.2012.06.710

[bib29] Panahi R. Kazemi S. & & Rezaie A. (2012). The relationship between learning styles and academic achievement: The role of gender and academic discipline. *Developmental Psychology (Journal of Iranian Psychologists)*, 8(30), 189–196. (Original work published in Persian)

[bib30] Pazargadi M. & & Tahmasebi S. (2010). Learning styles and their application in nursing. *Iranian Journal of Educational Strategies*, 3(2), 73–76. (Original work published in Persian)

[bib31] Shinnick M. A. & & Woo M. A. (2013). The effect of human patient simulation on critical thinking and its predictors in prelicensure nursing students. *Nurse Education Today*, 33(9), 1062–1067. 10.1016/j.nedt.2012.04.004 22564925

[bib32] Shirazi F. Sharif F. Molazem Z. & & Alborzi M. (2016). Dynamics of self-directed learning in M.Sc. nursing students: A qualitative research. *Journal of Advances in Medical Education & Professionalism*, 5(1), 33–41. PMC5238494 28124019

[bib33] Taghavi Larijani T. Mardani Hmouleh M. Rezaei N. Ghadiriyan F. & & Rashidi A. (2014). Relationship between assertiveness and critical thinking in nursing students. *Journal of Nursing Education*, 3(1), 32–40. (Original work published in Persian)

[bib34] Tulbure C. (2012). Learning styles, teaching strategies and academic achievement in higher education: A cross-sectional investigation. *Procedia-Social and Behavioral Sciences*, 33, 398–402. 10.1016/j.sbspro.2012.01.151

[bib35] Wangensteen S. Johansson I. S. Björkström M. E. & & Nordström G. (2010). Critical thinking dispositions among newly graduated nurses. *Journal of Advanced Nursing*, 66(10), 2170–2181. 10.1111/j.1365-2648.2010.05282.x 20384637 PMC2984541

[bib36] Zainol Abidin M. J. Rezaee A. A. Abdullah H. N. & & Singh K. K. B. (2011). Learning styles and overall academic achievement in a specific educational system. *International Journal of Humanities and Social Science*, 1(10), 143–152.

